# Prokineticin System Is a Pharmacological Target to Counteract Pain and Its Comorbid Mood Alterations in an Osteoarthritis Murine Model

**DOI:** 10.3390/cells12182255

**Published:** 2023-09-12

**Authors:** Giulia Galimberti, Giada Amodeo, Giulia Magni, Benedetta Riboldi, Gianfranco Balboni, Valentina Onnis, Stefania Ceruti, Paola Sacerdote, Silvia Franchi

**Affiliations:** 1 Department of Pharmacological and Biomolecular Sciences, University of Milan, 20133 Milan, Italy; giulia.galimberti@unimi.it (G.G.); giada.amodeo@unimi.it (G.A.); giulia.magni@unimi.it (G.M.); benedetta.riboldi@unimi.it (B.R.); stefania.ceruti@unimi.it (S.C.); paola.sacerdote@unimi.it (P.S.); 2 Department of Life and Environmental Sciences, University of Cagliari, 09124 Cagliari, Italy; gbalboni@unica.it (G.B.); vonnis@unica.it (V.O.)

**Keywords:** prokineticins, osteoarthritis pain, neuroinflammation, anxiety, depression

## Abstract

Osteoarthritis (OA) is the most prevalent joint disease associated with chronic pain. OA pain is often accompanied by mood disorders. We addressed the role of the Prokineticin (PK) system in pain and mood alterations in a mice OA model induced with monosodium iodoacetate (MIA). The effect of a PK antagonist (PC1) was compared to that of diclofenac. C57BL/6J male mice injected with MIA in the knee joint were characterized by allodynia, motor deficits, and fatigue. Twenty-eight days after MIA, in the knee joint, we measured high mRNA of PK2 and its receptor PKR1, pro-inflammatory cytokines, and MMP13. At the same time, in the sciatic nerve and spinal cord, we found increased levels of PK2, PKR1, IL-1β, and IL-6. These changes were in the presence of high GFAP and CD11b mRNA in the sciatic nerve and GFAP in the spinal cord. OA mice were also characterized by anxiety, depression, and neuroinflammation in the prefrontal cortex and hippocampus. In both stations, we found increased pro-inflammatory cytokines. In addition, PK upregulation and reactive astrogliosis in the hippocampus and microglia reactivity in the prefrontal cortex were detected. PC1 reduced joint inflammation and neuroinflammation in PNS and CNS and counteracted OA pain and emotional disturbances.

## 1. Introduction

Osteoarthritis (OA) is a complex disease of the whole joint and the major cause of joint pain and disabilities [1,2]. Pain in OA was generally considered a peripherally mediated state; however, a subset of OA patients also manifests centrally driven pain characteristics [3]. Thus, OA can be thought of as a “mixed” pain state that requires a tailored therapeutic approach. The slow breakdown of cartilage in the joint occurs in the presence of changes in the subchondral bone, synovium, and muscles. Considering that the cartilage is not innervated, OA pain is probably due to changes in the underlying bone and synovial inflammation [2]. Recent research revealed the crucial role of synovial inflammation in both OA incidence and progression and also in the genesis of pain [4,5,6]. Joint cells, such as synoviocytes, inflammatory cells (mast cells and macrophages), and chondrocytes, as well as infrapatellar fat pads that produce chemokines, cytokines, and proteases, which can initiate and maintain synovial inflammation, contribute to cartilage damage and sensitize primary afferent fiber terminals in adjacent tissues [6,7,8,9]. The increase in the nociceptive input from the periphery results in central sensitization in the dorsal horn of the spinal cord, where neurons become hyperexcitable [7], thus reducing their thresholds and enhancing their responses. Neurons begin to show increased responses to stimuli applied to regions adjacent to and remote from the joint, and the total receptive field can exhibit an enlargement. These changes represent the neuronal basis of primary hyperalgesia (at the site of disease) and secondary hyperalgesia (in areas adjacent to and remote from the joint) [7].

It is now recognized that OA pain is frequently accompanied by comorbid affective manifestations, such as anxiety and depression [10,11,12], which could additionally impact a patient’s quality of life [13,14] and aggravate pain perception, contributing to chronic pain establishment. The relationship between OA pain and mood disorders was also recently reported in a few preclinical studies using monosodium-iodoacetate (MIA)-induced OA in which MIA-treated animals exhibit psychiatric-like features [15,16,17,18,19]. Literature suggests that long-term pain triggers neural changes responsible for affective and cognitive alterations [20,21,22]. In addition, the relationship between pain and psychiatric disorders is demonstrated by the presence of a neurobiological overlapping in their molecular pathways, with a key role in neuroinflammation [23,24]. Indeed, in brain areas, increased levels of pro-inflammatory molecules, including cytokines released by reactive microglia and astrocytes, can participate in maladaptive neural reorganization, contributing to both sensory and affective components of chronic pain [23,25]. Moreover, an increase in supraspinal pro-inflammatory cytokines such as IL-6 and TNFα is often associated with anxiety and depression in patients [26,27,28].

Among cytokines, prokineticins (PKs) were recently identified as important mediators in inflammation and pain [29]. The chemokine PK family includes two ligands, prokineticin (PK) 1 and PK2 (or Bv8, bombina variegata 8 KDa), and two G protein-coupled receptors, named PKR1 and PKR2. PK2 is found to be upregulated in inflamed tissues, promoting inflammation and nociceptive sensitization [30,31]. Both PKs and PKRs are widely distributed in human/animal tissues and are involved in physiological activities [30]. PK system members are expressed by endothelial and immune cells but also by neurons and glial cells in stations involved in pain processing and participate in pain sensitization processes [30]. At the joint level, different cells may express PKRs and produce PK2 [32,33]. Synovial cells, including synovial fibroblasts and inflammatory cells such as macrophages and infiltrating granulocytes, are certainly important sources of PK2. The role of PK2 in the pathogenesis of different forms of arthritis was studied in both preclinical models and in humans. In a mouse model of collagen-induced arthritis (CIA), PK2 and PKR2 gene expression was significantly elevated in the joint, and this overexpression correlated with arthritis severity [32]. In addition, a very recent paper [34], using the same CIA model, also suggests that the administration of the PKRs antagonist PC1 improved clinical signs of arthritis, lowered plasma malondialdehyde (MDA) levels, and reduced joint expression of PK2, PKRs, TNFα, IL-1β, CD4, CD8, and NF-kB. In addition, both PK2 and PKRs were found to be highly expressed in the synovial tissue of rheumatoid arthritis (RA) and OA patients [33], and we recently described high PK2 levels in the synovial fluid of OA patients similar to those measured in the fluid of knee traumatic patients [35].

Interestingly, a role for PK2 in mood regulation [36,37] was also recently suggested. The intracerebroventricular injection of PK2 in mice leads to an increase in anxiety- and depression-like behavior, while mice deficient in PK2 gene display reduced anxiety and depression [36]. In addition, we recently demonstrated that chronic pain affects the development of mood alterations in chemotherapy-induced neuropathic pain models [37]. A long-lasting pain condition associated with bortezomib treatment was indeed correlated to the presence of anxiety and depression in mice, and these behavioral changes were accompanied by increased levels of PK2 and PKR2 in the hippocampus and by a general neuroinflammatory condition in the hippocampus, prefrontal cortex, and hypothalamus. The treatment with PKR antagonist PC1, contrasting pain and reducing the mRNA levels of neuroinflammatory markers, prevented the development of depression as well [37]. 

Based on this evidence, the antagonism of the PK system seems to be a strategic therapeutic approach to counteract local inflammatory and central sensitization processes as well as mood alterations associated with OA pain. In this work, we used the MIA-OA model that, by inducing functional impairment resembling human OA, represents one of the most validated and used rodent models to assess pain and its treatment [38,39]. In the present paper, we did not limit our research to the affected joint [32,34], but we dissected the role of PKs from the joint up to the spinal cord and supraspinal areas as well. The role of PK system activation in joint inflammation and in neuroinflammation in the PNS and CNS (i.e., peripheral sciatic nerve, spinal cord, and supraspinal areas) was evaluated in relation to the presence of sensory hypersensitivity as well as anxiety- and depression-like behavior in mice. The effect of PC1, a PK system antagonist, on pain, neuroinflammation, and mood alterations was compared to that of diclofenac, a nonsteroidal anti-inflammatory drug (NSAID) commonly used for OA pain management.

## 2. Materials and Methods

### 2.1. Animals

In all experiments, 10-week-old C57BL/6J male mice (Charles River Laboratories, Calco, Italy) were used (n = 84). Animals were acclimatized for two weeks before starting the procedures. During the entire experimental protocol, animals were housed with standard environmental conditions (12 h light/dark cycle at 22 ± 1 °C and humidity of 55 ± 10%) and fed with dry pellets and water ad libitum. Mice were housed in three or four per cage (Macrolon type II cages—26 cm × 20 cm × 14 cm), each equipped with chipboard litter, nesting material, and environmental enrichments. During the housing, animals were subjected to periodic veterinary checks. All experimental procedures were conducted in accordance with the guidelines issued by the Animal Care and Use Committee of the Italian Ministry of Health (DL 116/92 e DL111/94-B; Authorization 180/2020 to PS) and with the directives of the European Community which regulate animal research (86/609/EEC). The 3Rs (Re-place, Reduce, Refine) principle has been adopted and respected, trying to minimize the number of animals used and their suffering. 

### 2.2. Induction of Osteoarthritis

Mice were randomized into two experimental groups, using a computerized method for randomization, in order to be treated with either saline solution (control, CTR mice, n = 42) or monosodium iodoacetate (MIA, Sigma-Aldrich, Milan, Italy) for osteoarthritis induction (MIA mice, n = 42). Under general anesthesia (i.p. 60 mg/kg sodium pentobarbital, Sigma-Aldrich, Milan, Italy), MIA mice were treated with a single intra-articular (IA) injection of 1 mg of MIA in a volume of 10 μL of saline solution (NaCl 0.9%) [40,41]. CTR mice received an IA injection of saline solution (10 µL). For both groups, IA administration was performed in the right knee. 

### 2.3. Pharmacological Treatments

After 14 days from OA induction, CTR and MIA mice were divided into three experimental groups (n = 14/each) and started the treatment with either drugs or vehicle. In detail, both CTR and MIA mice were treated with vehicle (saline solution, 10 µL/g), with the non-peptidic antagonist of the PK system PC1 (s.c., 150 µg/kg, twice a day) [37,42,43] or with the NSAID diclofenac (i.p., 10 mg/kg, once a day, Novartis, Milan, Italy) [44]. All drugs were freshly prepared, and each treatment was daily (once/twice) administered for 14 days (from day 14 until day 27). Mice were sacrificed the day after the last drug administration (day 28). 

### 2.4. Behavioral Tests

Animals in the different experimental groups were divided into two cohorts [45], i.e., anxiety or depression cohort, in order to avoid performing too many tests on the same animal. Mice belonging to the anxiety cohort were tested for pain (n = 7), while those of the depression cohort were tested for locomotor activity and fatigue (n = 7). Mechanical allodynia (von Frey test) and locomotor activity (Rotarod test) were tested before (0, basal) and 7, 14, 21, 28 days post-OA-induction. Fatigue-like behavior (Treadmill test) was assessed before (0, basal) and 14 and 28 days post-OA-induction. All tests were performed before the (first) daily drug treatment. Additionally, the antiallodynic effect of both PC1 and diclofenac was also evaluated acutely: 30, 60, 90, 120, 150, 180, and 210 min after the first drug injection, performed at day 14 post-OA-induction. At the end of the experimental protocol, 28 days post-OA-induction, corresponding to 14 days of chronic pharmacological treatment, both anxiety- and depression-like behaviors were evaluated in mice. All tests were performed in the morning, before the (first) daily drug administration. Animals were acclimatized to the new environment for 30 min, and evaluations were executed by blinded and trained researchers.

#### 2.4.1. Mechanical Allodynia: Von Frey Test

Mechanical allodynia was evaluated through the von Frey test using the dynamic plantar aesthesiometer (Ugo Basile, Italia). Animals were placed in a plexiglass cage (w 8.5 cm × h 8.5 cm) over a metallic grid, and a mechanical stimulus through the von Frey filament (metal filament, 0.5 mm diameter, ranging up to 10 g in 10 s) was applied in the middle plantar surfaces of hind paws. The test was conducted in both contra- (data not shown) and ipsi-lateral paw. The test started with a value of force below the detection threshold and continued with an increased force until the animal removed its paw [41,43]. Response to mechanical stimuli (Paw Withdrawal Threshold, PWT) was expressed in grams (g).

#### 2.4.2. Locomotor Function

For three consecutive days before OA induction, mice were trained on the Rotarod and Treadmill apparatus (Ugo Basile, Gemonio, Italy).

Rotarod test: locomotor activity was evaluated by an accelerating protocol: 4–20 rpm in 600 s. The test was considered finished when the animal fell down (fall time), arrived at the cut-off (600 s) or if the animal stood still and completed three full passive rotations (last rotation time) [41]. 

Treadmill test: the fatigue-like behavior was assessed with the treadmill apparatus (Ugo Basile, Gemonio, Italy) with a +10° inclination, according to the following protocol: a session in acceleration 3–20 m/min for 15 min, followed by a session at a constant speed of 20 m/min until the cut-off (120 min) or exhaustion (5 s in the fatigue zone, i.e., the final part (1/3) of the running lane) [46].

#### 2.4.3. Anxiety-like Behavior

Light/dark box test: the apparatus is made of a square arena divided into two chambers communicating through a slot (5 × 5 cm). One room has black walls and is not exposed to light (12 l × 40 d × 50 h cm, 1/3 of the box); the other has white walls and is exposed to light (24 l × 40 d × 50 h cm, 2/3 of the box). During the test session, each animal was located in the light chamber and allowed to freely move between the two chambers for 5 min, and the total time spent in the light chamber (s) was measured. Usually, an anxious mouse shows a decrease in this parameter [37,45].

Elevated plus maze test: the apparatus (Ugo Basile, Gemonio, Italy) is composed of two arms without walls (open arms, 40 cm each) and two with walls (closed arms, 40 cm each) linked by a central zone and elevated from the floor (60 cm). During the test session, the mouse was placed in the middle of the 4 arms and left free to explore for 5 min, and the total time spent in the open arms (s) was measured: anxiety-like behavior is linked to a decrease in exploratory activity in open arms [47]. 

#### 2.4.4. Depression-like Behavior

The mouse was gently placed in a 3 L glass beaker (h 27 cm, Ø 14.5 cm) filled with water (23 ± 2 °C) and allowed to swim for 6 minutes. The parameters under analysis were the latency time (first time of prolonged immobility, >10 s), expressed in seconds, and the total immobility time during the last 4 min (expressed as % over 240 s) [37,45]. At the end of the test, the animal was dried and put back in its cage.

### 2.5. Tissue Collection

At the end of the experimental protocol, i.e., after 28 days post-OA-induction and after 14 days of treatment with PC1 or diclofenac, mice were sacrificed by decapitation. Right hind leg was harvested immediately after sacrifice, the skin and muscle removed, and the knee joint isolated by sharp dissection through the growth plates [48]. Ipsilateral sciatic nerve, spinal cord (L3–L5), and brain areas involved in mood regulation (e.g., hippocampus and prefrontal cortex) were isolated and collected according to standardized laboratory procedures [37,49]. All tissues were frozen in liquid nitrogen and stored at −80 °C until biochemical evaluations.

#### 2.5.1. RT-qPCR

RNA was extracted using TRIzol^®^ reagent (Invitrogen, Waltham, MA, USA) according to the manufacturer’s instructions. Briefly, after adding TRizol, tissues were homogenized using an ultrasonic processor (UP50H DR. Hielscher, Teltow, Germany) for the sciatic nerve or Ultra Turrax (T25 Janke & Kunkel- IKA Labor Technik, Staufen, Germany) for all the other tissues. The whole knee joint was initially reduced to small pieces with scissors before being processed with homogenizer. cDNA was obtained from RNA using the reverse transcriptase kit LunaScript™ (BioLabs, London, UK) and used as template in quantitative PCR (QuantStudio5™). Real-time PCR was performed using Luna^®^ Universal Probe, qPCR Master Mix (BioLabs, London, UK), and specific TaqmanTM Gene expression assays (Thermofisher Scientific, Waltham, MA, USA): Prokineticin 2 (PK2: Mm01182450_g1), prokineticin receptors (PKR1: Mm00517546_m1 and PKR2: Mm00769571_m1), interleukin-1β (IL-1β: Mm00434888_m1), interleukin 6 (IL-6: Mm00446190_m1), tumor necrosis factor-α (TNFα: Mm00443258_m1), glial fibrillary acidic protein (GFAP: Mm01253033_m1), cluster of differentiation 11b (CD11b: Mm00434455_m1), ionized calcium-binding adapter molecule 1 (Iba1: Mm00479862_g1), activating transcription factor 3 (ATF3: Mm00476033_m1) and matrix metalloprotein-ase13 (MMP13: Mm00439491_m1). The mRNA levels of each gene were normalized to GAPDH (Mm99999915_g1), and data were analyzed using the 2^−∆∆CT^ method. Each sample was run in triplicate.

#### 2.5.2. ELISA Assay

TNFα protein levels were evaluated in hippocampus and prefrontal cortex homogenates using ELISA assay according to the manufacturer’s instructions (Invitrogen™, Thermofisher Scientific, Waltham, MA, USA). For protein extraction, tissues were homogenized in 500 µL ice-cold PBS supplemented with 0.05% EDTA and 0.5% protease inhibitor cocktail (Roche Diagnostics, Monza, Italy). The homogenates were centrifuged at 13,000 rpm for 15 min at 4 °C, and supernatants collected. The total protein content was measured with Lowry’s method [41,43]. For each sample, the ratio between cytokine content (pg) and total protein content (mg) was calculated. 

### 2.6. Immunohistochemistry and Image Analysis 

Mice were deeply anesthetized (60 mg/kg of sodic pentobarbital, i.p.) and transcardially perfused with PBS, followed by 4% formalin. After sacrifice, the brains were excised, post-fixed in 10% formalin for 60 min, cryoprotected in 30% sucrose (48 h), embedded in OCT mounting medium (VWR, Milano, Italy), and cut coronally on a cryostat at 20 μm thickness at the level of the prefrontal cortex and hippocampus. Sections were incubated for 45 min in PBS containing 10% normal goat serum (Life Technologies, Monza, Italy) and 0.3% Triton X-100 (Sigma–Aldrich, Merck group), and then overnight at RT with the following primary antibodies: rabbit antibodies against the microglia marker Iba1 (anti-Iba1, 1:500; Wako, Richmond, VA, USA) or the astrocyte marker GFAP (anti-GFAP, 1:600; Dako, Milan, Italy). Sections were then rinsed three times with PBS and incubated for 1 h RT with AlexaFluor^®^ 488-conjugated secondary antibody (1:600; Life Technologies). All antibodies were diluted in PBS containing 0.3% Triton X-100 and 5% normal goat serum. Cell nuclei were counterstained with the Hoechst33258 dye (1:20,000; Sigma-Aldrich, Merck group). Samples were examined with Zeiss Axioskop fluorescent microscope (Carl Zeiss, Milan, Italy) with the aid of the NIH ImageJ software, as described below. The number of Iba1+ cells was counted in discrete brain areas (i.e., prefrontal cortex and hippocampus) by a blinded researcher, acquired at 20X magnification, and expressed as number of positive cells/area in mm^2^. To better analyze glial cell morphology and to evaluate morphological signs of activated microglia and astrocytes, Iba1- and GFAP-immunostained images of sections acquired at 20× magnification were converted to binary grayscale, and the average cell size was evaluated by the “Particle analysis” tool of the Fiji-ImageJ software [50]. Immunostaining negative controls were performed in the prefrontal cortex and hippocampus. Primary antibodies (anti-Iba1 and anti-GFAP) were omitted, and sections were incubated with secondary antibody (AlexaFluor^®^ 488-conjugated goat anti-rabbit) only.

### 2.7. Statistical Analysis 

Data are reported as the mean ± SEM of 3–7 mice/group. In detail, 3 mice/group for immunohistochemistry analysis, 5 mice/group for ELISA assay, 6 mice/group for RT-qPCR, and 7 mice/group for behavioral tests. ANOVA analysis of variance was used to analyze pain, locomotor activity (Two-way), and mood results (One-way); Bonferroni’s post hoc test was then applied. One-way ANOVA, followed by Tukey’s test, was used for the biochemical analysis. Statistical analysis was performed using GraphPad Prism 9 (San Diego, CA, USA). For all analyses, differences were considered significant at *p* ≤ 0.05.

## 3. Results

### 3.1. Mechanical Allodynia, Locomotor Activity and Fatigue-like Behavior in MIA OA Mice. Effect of PC1 and Diclofenac

**Figure 1:**
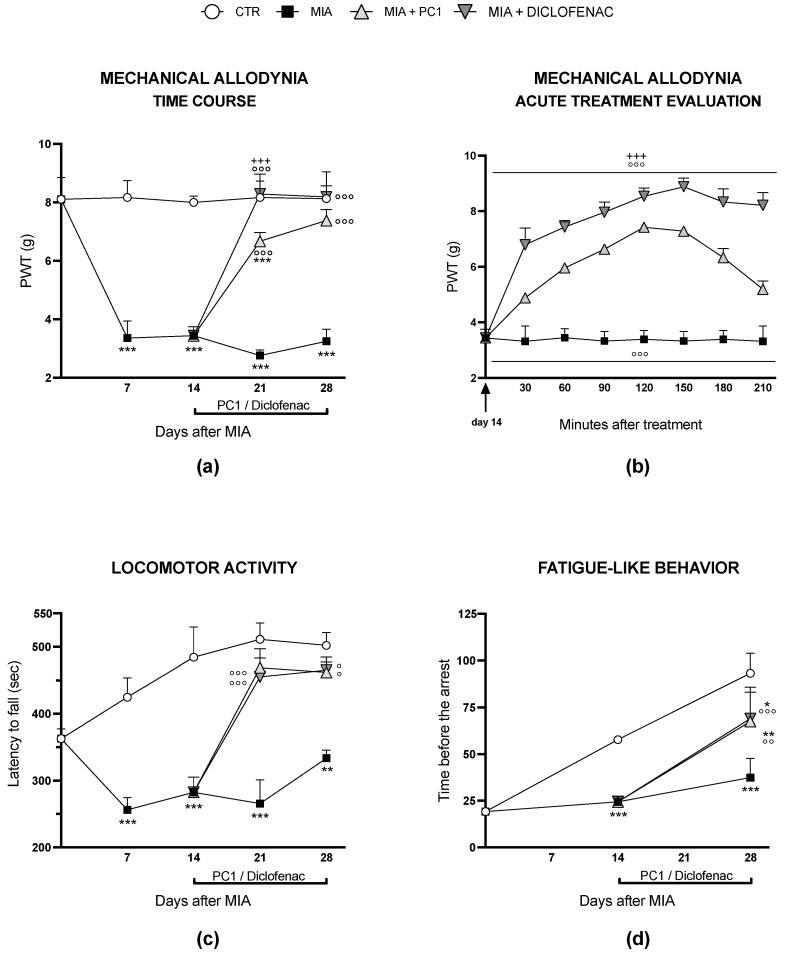
Effect of PC1 and diclofenac on mechanical allodynia and motor performance in OA mice. Osteoarthritis (OA) was induced in mice by intra-articular administration of monosodium iodoacetate (MIA, 1 mg in 10 µL of saline, right knee). After 14 days from OA induction, the chronic treatment (starting from day 14) with PC1 (150 µg/kg s.c., twice a day) or diclofenac (10 mg/kg i.p., once a day) was started. Mechanical allodynia was evaluated by von Frey test every 7 days from day 0 to 28 (**a**), and acutely after the first PC1 and diclofenac administration (on day 14) with repeated measures every 30 min up to 210 min (**b**). Motor performance was assessed by both Rotarod test (from day 0 to 28 every 7 days; (**c**), and treadmill test (from day 0 to 28, every 14 days; (**d**). Results are expressed as mean ± SD (**a**,**b**) or SEM (**c**,**d**) of 7 animals/group. Statistical analysis was performed by Two-way ANOVA followed by Bonferroni’s post hoc test. * *p* < 0.05, ** *p* < 0.01, *** *p* < 0.001 vs. CTR; ° *p* < 0.05, °° *p* < 0.01, °°° *p* < 0.001 vs. MIA; +++ *p* < 0.001 vs. MIA + PC1. Time x treatments: (**a**) F (12,288) = 83.62, *p* < 0.0001; (**b**) F (14,144) = 33.95, *p* < 0.0001; (**c**) F (12,288) = 5.914, *p* < 0.0001; (**d**) F (6,198) = 9.517, *p* < 0.0001.

As shown in Figure 1a, MIA mice are characterized by the development of mechanical allodynia that is already evident 7 days after MIA administration and is maintained thereafter until day 28 (*p* < 0.001). Diclofenac completely counteracts allodynia after seven days of chronic treatment (i.e., at day 21 after MIA, *p* < 0.001), and its effect remains maximal after 14 days of treatment (day 28 after MIA). PC1 also significantly counteracts allodynia after 7 days of chronic treatment (day 21 after MIA, *p* < 0.001), bringing PWT back to control levels at the end of the treatment (day 28 after MIA). Panel 1b shows the time course of the acute antiallodynic effect of the two drugs, measured after the first drug administration, performed 14 days post-OA-induction. Both PC1 and diclofenac display a rapid antiallodynic effect that is evident 30 min after the acute injection (*p* < 0.001) and is still present at 210 min (*p* < 0.001). Even in the presence of similar efficacy, the antiallodynic effect of diclofenac appears to be greater than that of PC1 and is maintained at the maximal level for a longer period of time, up to 210 min post-drug administration (*p* < 0.001). The locomotor activity of MIA mice (panel 1c) is significantly lower compared to CTR mice for the whole experimental duration (*p* < 0.01). PC1 and diclofenac similarly improve mice performance (*p* < 0.001 vs. MIA) after 7 days of repetitive treatment (i.e., day 21 after MIA), and the effect of the two drugs is also similar after 14 days of treatment (i.e., day 28 after MIA). Panel 1d reports the results of the Treadmill test. As expected, the performance of CTR mice, evaluated as the time before the arrest during the treadmill session, improves over time, thus showing how CTR animals become more resistant to fatigue. MIA mice were unable to improve their motor performance, a behavior that can be attributed to the presence of a motor deficit due to OA. The chronic treatment with either PC1 or diclofenac is able to counteract this symptom, improving mice performance (*p* < 0.01 vs. MIA).

### 3.2. Anxiety- and Depression-like Behavior in MIA OA Mice. Effect of PC1 and Diclofenac

**Figure 2:**
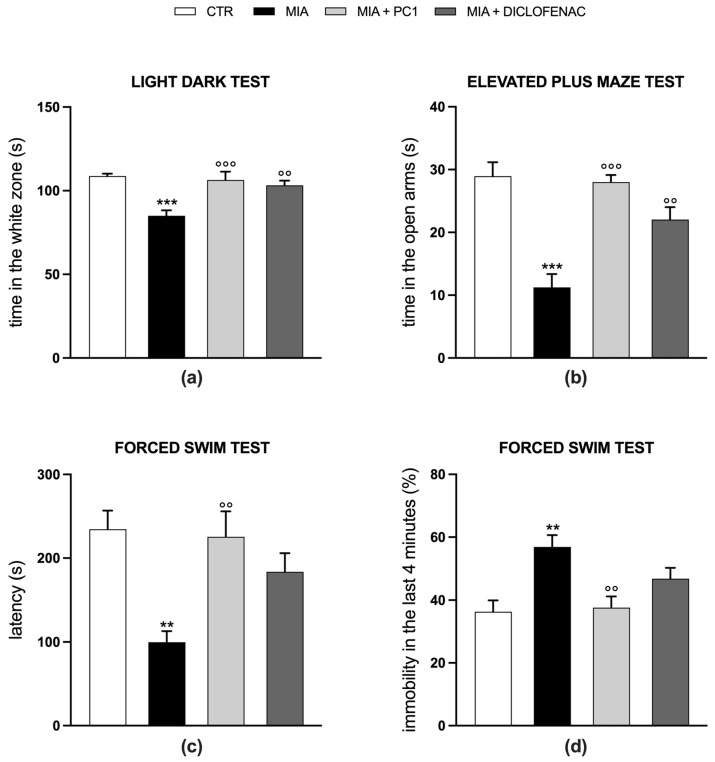
Effect of PC1 and diclofenac on anxiety- and depression-like behavior in OA mice. Mood alterations were evaluated at the end of the experimental protocol (day 28 post-OA-induction). Anxiety-like behavior was evaluated with the light-dark box test, considering the time spent in the white zone (s) (**a**) and with the elevated plus maze test, considering the time spent in the open arms (s) (**b**). Depression-like behavior was evaluated with forced swim test, considering both the latency (**c**) and the immobility time in the last 4 min of the test (**d**). Results are expressed as mean ± SEM of 7 animals/group. Statistical analyses were performed by One-way ANOVA followed by Bonferroni’s post hoc test. ** *p* < 0.01, *** *p* < 0.001 vs. CTR; °° *p* < 0.01, °°° *p* < 0.001 vs. MIA. Time x treatments: (**a**) F (3,24) = 10.46, *p* = 0.0001; (**b**) F (3,24) = 18.31, *p* < 0.0001; (**c**) F (3,24) = 7.196, *p* = 0.0013; (**d**) F (3,24) = 7.096, *p* = 0.0014.

Figure 2 shows the results regarding anxiety- and depression-like behaviors. Twenty-eight days after MIA injection, in the presence of a well-established hypersensitivity condition (see Figure 1), MIA mice display anxiety- (panels a and b) and depression-like behaviors (panels c and d). Indeed, MIA mice spend less time in the white chamber of the light-dark box (*p* < 0.001) and in the open arms (*p* < 0.001) of the elevated plus maze in comparison to CTR mice, as shown in panels a and b, respectively, showing an anxiety-like behavior.

Furthermore, as observed in the forced swim test (panels c and d), MIA mice are characterized by a decrease in the latency time (panel c; *p* < 0.01), corresponding to the first time of arrest, and an increase in the immobility time in the last 4 min of testing (panel d; *p* < 0.01), suggesting a depression-like status. Fourteen days of PC1 treatment is able to contrast or prevent both the development of anxiety- (panels a and b; *p* < 0.001) and depression-like behaviors (panels c and d; *p* < 0.01). The treatment with diclofenac significantly contrasts only anxiety (panels a and b, *p* < 0.01 vs. MIA), while it seems less effective on depression-like behavior (panels c and d; *p* > 0.05 vs. MIA). No behavioral effect of either PC1 or diclofenac was observed in CTR mice (data not shown).

### 3.3. PK System, Cytokines, and MMP in MIA OA Mice Knee Joint. Effect of PC1 and Diclofenac

**Figure 3:**
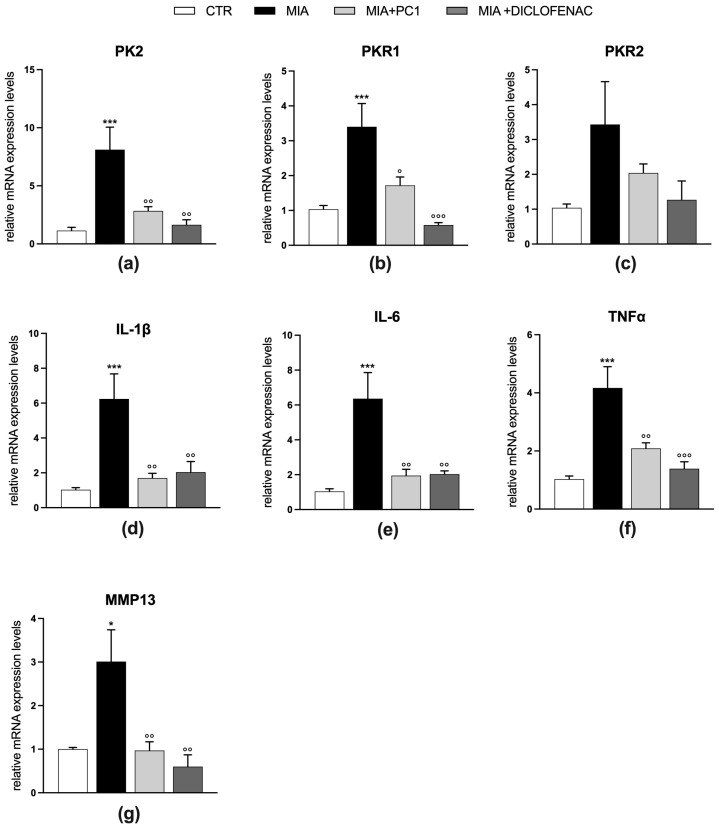
Effect of PC1 and diclofenac on the expression of PK system members and of inflammatory/degenerative markers in OA knee joint. mRNA expression levels (qRT-PCR) of the PK system members PK2 (**a**), PKR1 (**b**) and PKR2 (**c**), and of the pro-inflammatory cytokines IL-1β (**d**), IL-6 (**e**) and TNFα (**f**) and MMP-13 (**g**) were evaluated in the knee joint (day 28 post-OA). Results are normalized on the housekeeping gene GAPDH and expressed as fold over control group. Data are the mean ± SEM of 6 animals/group. Statistical analyses were performed by One-way ANOVA followed by Tukey’s post hoc test. * *p*< 0.05, *** *p* < 0.001 vs. CTR; ° *p* < 0.05, °° *p* < 0.01, °°° *p* < 0.001 vs. MIA. Treatments: (**a**) F (3,20) = 9.816, *p* = 0.0003; (**b**) F (3,20) = 11.70, *p* = 0.0001; (**c**) F (3,20) = 2.469, *p* = 0.0916; (**d**) F (3,20) = 8.813, *p* = 0.0006; (**e**) F (3,20) = 9.339, *p* = 0.0005; (**f**) F (3,20) = 12.37, *p* < 0.0001; (**g**) F (3,20) = 7.367, *p* = 0.0016.

As shown in Figure 3, MIA mice are characterized by an inflammatory condition in the OA knee joint where, 28 days after MIA injection, a significant upregulation of both PK2 and PKR1 expression is detected (panels a and b; *p* < 0.001), while PKR2 levels are not significantly affected (panel c). 

In parallel, the mRNA expression level of the pro-inflammatory cytokines IL-1β (panel d), IL-6 (panel e), and TNFα (panel f) are also increased (*p* < 0.001). In the presence of this consistent inflammatory condition, the matrix metalloproteinase MMP13 mRNA appears upregulated (panel g; *p* < 0.05), suggesting an active catabolic activity on the cartilage. Chronic treatment with both PC1 and diclofenac counteracts this condition by normalizing the PK system (panels a and b; *p* < 0.05) and reducing pro-inflammatory cytokines (panels d–f; *p* < 0.01) and MMP13 (panel g; *p* < 0.01) levels.

### 3.4. PK System, Cytokines, and Glial Marker Evaluation in the Sciatic Nerve and Spinal Cord of MIA OA Mice. Effect of PC1 and Diclofenac

**Figure 4:**
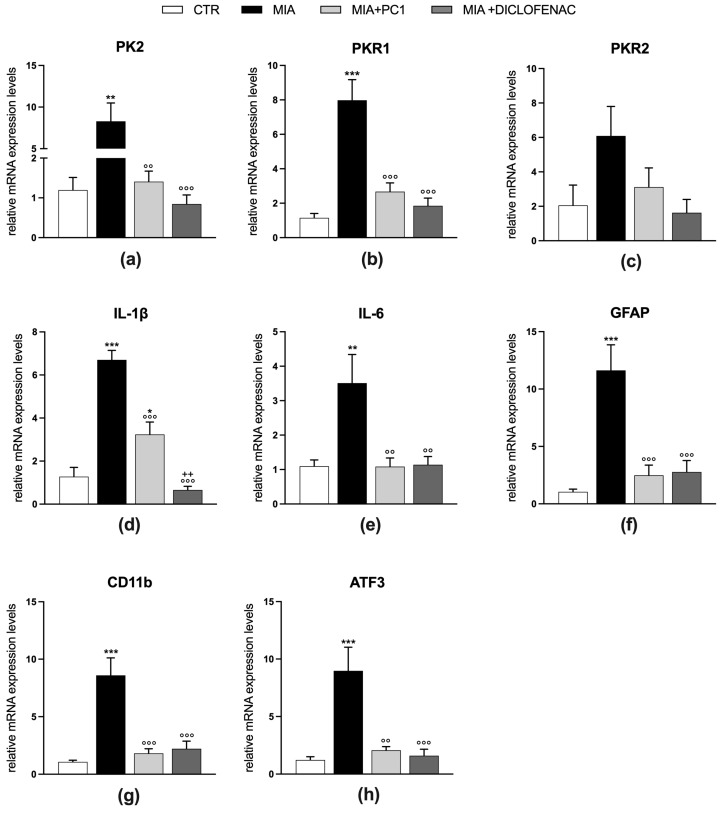
Effect of PC1 and diclofenac on the expression of PK system members and neuroinflammatory markers in the sciatic nerve of OA mice. mRNA expression levels (qRT-PCR) of PK members PK2 (**a**), PKR1 (**b**) and PKR2 (**c**), of the pro-inflammatory cytokines IL-1β (**d**), IL-6 (**e**), of the glial markers GFAP (**f**) and CD11b (**g**) and of the neuronal damage marker ATF3 (**h**) were evaluated in the sciatic nerve (day 28 post-OA). Results are normalized on the housekeeping gene GAPDH and expressed as fold over control group. Data are the mean ± SEM of 6 animals/group. Statistical analyses were performed by One-way ANOVA followed by Tukey’s post hoc test. * *p* < 0.05, ** *p* < 0.01, *** *p* < 0.001 vs. CTR; °° *p* < 0.01, °°° *p* < 0.001 vs. MIA; ++ *p* < 0.01 vs. MIA + PC1. Treatments: (**a**)F (3,20) = 10.10, *p* = 0.0003; (**b**) F (3,20) = 12.18, *p* < 0.0001; (**c**) F (3,20) = 2.628, *p* = 0.0784; (**d**) F (3,20) = 40.33, *p* < 0.0001; (**e**) F (3,20) = 6.829, *p* = 0.0024; (**f**) F(3,20) = 13.65, *p* < 0.0001; (**g**) F (3,20) = 16.38, *p* < 0.0001, (**h**) F (3,20) = 11.56, *p* = 0.0001.

**Figure 5:**
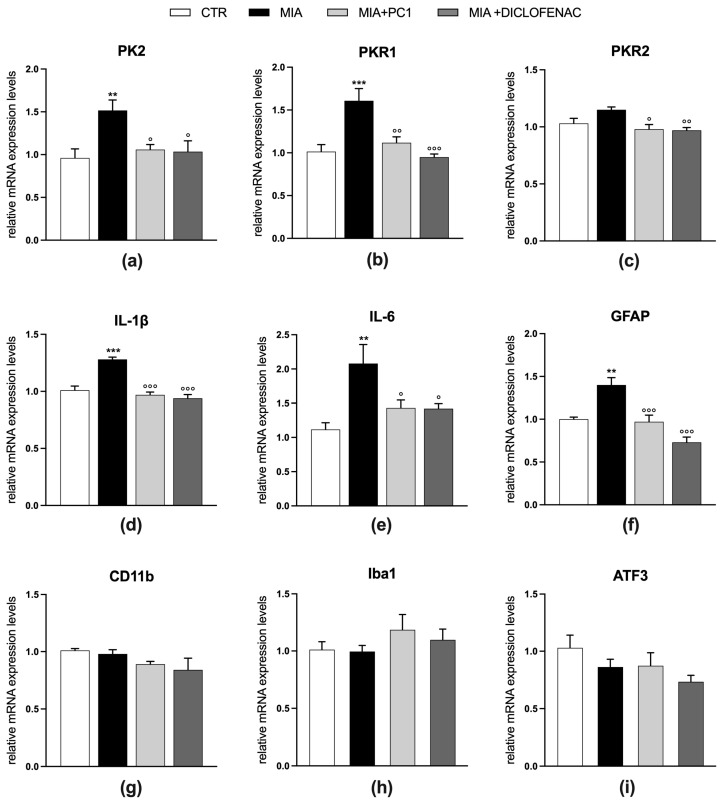
Effect of PC1 and diclofenac on the expression of PK system members and neuroinflammatory markers in the spinal cord of OA mice. mRNA expression levels (qRT-PCR) of the PK members PK2 (**a**), PKR1 (**b**) and PKR2 (**c**), of the pro-inflammatory cytokines IL-1β (**d**), IL-6 (**e**), of the glial markers GFAP (**f**), CD11b (**g**) and Iba1 (**h**), and of the neuronal damage marker ATF3 (**i**) were evaluated in the L3-L5 spinal cord portion (day 28 post-OA). Results are normalized on the housekeeping gene GAPDH and expressed as fold over control group. Data are the mean ± SEM of 6 animals/group. Statistical analyses were performed by One-way ANOVA followed by Tukey’s post hoc test. ** *p* < 0.01, *** *p* < 0.001 vs. CTR; ° *p* < 0.05, °° *p* < 0.01, °°° *p* < 0.001 vs. MIA. Treatments: (**a**) F (3,20) = 5.584, *p* = 0.0060; (**b**) F (3,20) = 10.78, *p* = 0.0002; (**c**) F (3,20) = 5.590, *p* = 0.0060; (**d**) F (3,20) = 28.35, *p* < 0.0001; (**e**) F (3,20) = 6.217, *p* = 0.0037; (**f**) F (3,20) = 17.41, *p* < 0.0001; (**g**) F (3,20) = 1.963, *p* = 0.1520; (**h**) F (3,20) = 0.8829, *p* = 0.4668; (**i**) F (3,20) = 1.767, *p* = 0.1859.

Twenty-eight days after MIA injection, mice are also characterized by the presence of a neuroinflammatory condition in the sciatic nerve (Figure 4) and in the spinal cord (Figure 5).

In these tissues, a significant upregulation of both PK2 (*p* < 0.01) and PKR1 (*p* < 0.001) expression (panels a and b, Figure 4 and Figure 5) is observed, which is accompanied by an increase in the pro-inflammatory cytokines IL-1β (*p* < 0.001) and IL-6 (*p* < 0.01; panels d and e, Figure 4 and Figure 5).

No significant alteration is evident for PKR2 mRNA levels (panel c, Figure 4 and Figure 5). In the sciatic nerve (Figure 4), these alterations are accompanied by increased mRNA levels of GFAP, which in peripheral nerves represents a marker of activated Schwann cells [51] (panel f; *p* < 0.001) and of the macrophage marker CD11b (panel g; *p* < 0.001), and by the increase in the expression levels of the marker of neuronal damage ATF3 (panel h, *p* < 0.001). Conversely, in the spinal cord of MIA mice (Figure 5), only the astrocytic marker GFAP is found upregulated (panel f; *p* < 0.01), while no alterations in the levels of microglia/macrophage markers CD11b (panel g) and Iba1 (panel h) as well as of ATF3 (panel i) are registered. PC1 and diclofenac similarly counteract neuroinflammation in both the sciatic nerve and spinal cord (Figure 4 and Figure 5, respectively), reducing the levels of all the altered parameters (*p* < 0.05).

### 3.5. PK System, Cytokines, and Glial Marker Evaluation in Brain Areas Involved in Mood Regulation: Hippocampus and Prefrontal Cortex. Effects of PC1 and Diclofenac

Twenty-eight days after MIA injection, OA mice showing anxiety- and depression-like behaviors are also characterized by supraspinal neuroinflammation in brain areas involved in mood regulation, such as the hippocampus and the prefrontal cortex.

#### 3.5.1. Hippocampus

**Figure 6:**
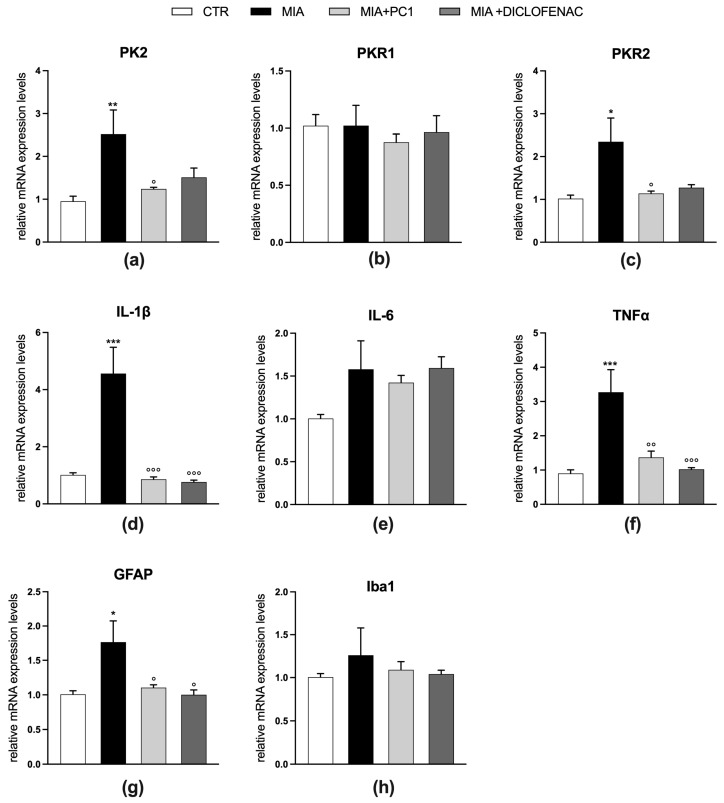
Effect of PC1 and diclofenac on the expression of PK system members and neuroinflammatory markers in the hippocampus. mRNA expression levels (qRT-PCR) of the PK system members PK2 (**a**), PKR1 (**b**) and PKR2 (**c**), pro-inflammatory cytokines IL-1β (**d**), IL-6 (**e**), and TNFα (**f**) and glial markers GFAP (**g**) and Iba1 (**h**) were evaluated in the hippocampus (day 28 post-OA). Results are normalized on the housekeeping gene GAPDH and expressed as fold over control group. Data are the mean ± SEM of 6 animals/group. Statistical analyses were performed by One-way ANOVA followed by Tukey’s post hoc test. * *p* < 0.05, ** *p* < 0.01, *** *p* < 0.001 vs. CTR; ° *p* < 0.05, °° *p* < 0.01, °°° *p* < 0.001 vs. MIA. Treatments: (**a**) F (3,20) = 4.883, *p* = 0.0105; (**b**) F (3,20) = 0.2795, *p* = 0.8396; (**c**) F (3,20) = 4.621, *p* = 0.0130; (**d**) F (3,20) = 15.84, *p* < 0.0001; (**e**) F (3,20) = 2.205, *p* = 0.1191; (**f**) F (3,20) = 10.20, *p* = 0.0003; (**g**) F (3,20) = 5.157, *p* = 0.0084; (**h**) F (3,20) = 0.4427, *p* = 0.7250.

**Figure 7:**
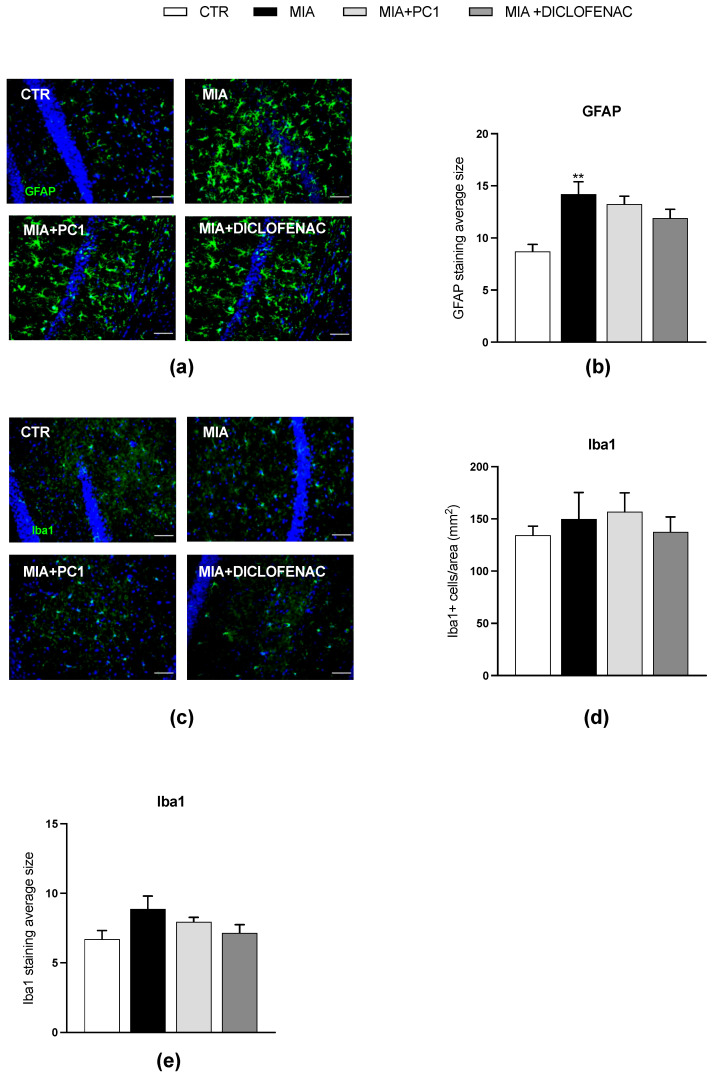
Reactive astrogliosis in the hippocampus of OA mice: immunohistochemical analysis. Images of hippocampal sections (day 28 post-OA) after IHC staining for GFAP (green; **a**) and Iba1 (green; **c**) have been converted to black and white and the “particle analysis” Fiji/ImageJ tool was then utilized to measure the average size of GFAP- and Iba1-positive particles (**b** and **e**, respectively). The number of Iba1-positive cells/area has also been evaluated (**d**). Nuclei were labeled with the Hoechst33258 dye (blue). Scale bars: 50 µm. Data are the mean ± SEM of 3 animals/group. Statistical analyses were performed by means of One-way ANOVA followed by Tukey’s post hoc test. ** *p* < 0.01 vs. CTR. Treatments: (**b**) F (3,8) = 7.381, *p* = 0.0108; (**d**) F (3,8) = 0.3070, *p* = 0.8199; (**e**) F (3,8) = 1.709, *p* = 0.2419.

As shown in Figure 6, in the hippocampus, we observed an upregulation of PK2 (panel a; *p* < 0.01) and PKR2 (panel c; *p* < 0.05) mRNA expression, together with an increase in the expression of IL-1β and TNFα (panels d and f; *p* < 0.001) and of the astrocytic marker GFAP (panel g; *p* < 0.05). The levels of PKR1 (panel b), IL-6 (panel e), and Iba1 (panel h) are unaffected by MIA injection. As shown in panels a and c, only PC1 significantly reduces PK2 and PKR2 expression (*p* < 0.05), despite a trend toward reduction after diclofenac. The two drugs similarly normalize the altered neuroinflammatory markers (panels d, f, and g; *p* < 0.05). As shown in Figure 7, immunofluorescence evaluations confirm qPCR data demonstrating reactive astrogliosis in the hippocampus of MIA mice (panels a and b), as shown by the increase in the average size of GFAP-positive cells (panels b; *p* < 0.01). Nevertheless, at variance from qPCR data, no significant protective effect of PC1 and diclofenac is detected. In addition, fully in line with qPCR results, no signs of microgliosis are observed in the hippocampus of MIA-treated mice (panels c–e). Supplementary Figure S1, panel a, shows immunostaining negative control.

#### 3.5.2. Prefrontal Cortex

**Figure 8:**
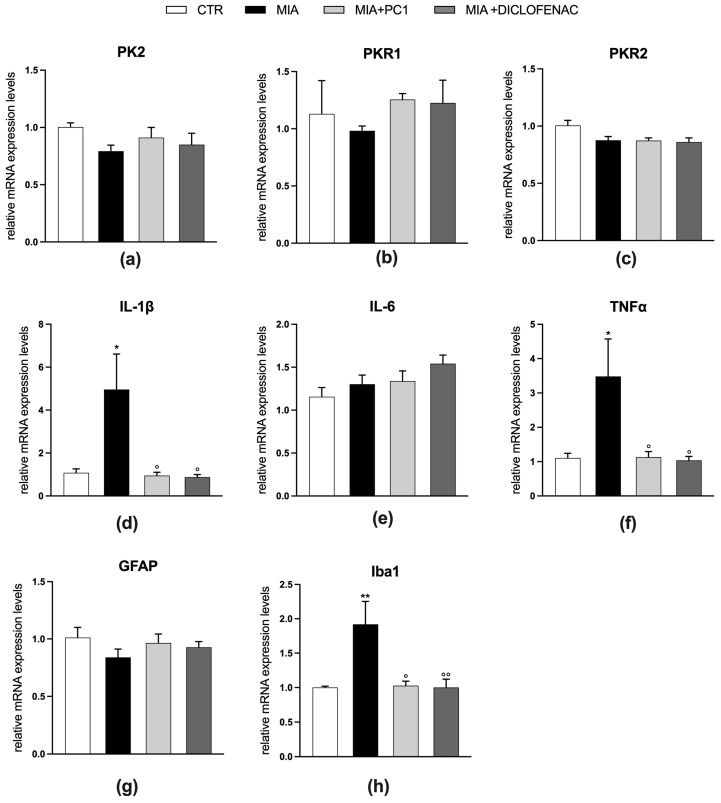
Effect of PC1 and diclofenac on the expression of PK system members and neuroinflammatory markers in the prefrontal cortex of OA mice. mRNA expression levels (qRT-PCR) of the PK system members PK2 (**a**), PKR1 (**b**) and PKR2 (**c**), of the pro-inflammatory cytokines IL-1β (**d**), IL-6 (**e**), and TNFα (**f**) and of the glial markers GFAP (**g**) and Iba1 (**h**) were evaluated in the prefrontal cortex (day 28 post-OA). Results are normalized on the housekeeping gene GAPDH and expressed as fold over control group. Data are the mean ± SEM of 6 animals/group. Statistical analyses were performed by One-way ANOVA followed by Tukey’s post hoc test. * *p* < 0.05, ** *p* < 0.01, vs. CTR; ° *p* < 0.05, °° *p* < 0.01 vs. MIA. Treatments: (**a**) F (3,20) = 1.499, *p* = 0.2453; (**b**) F (3,20) = 0.4740, *p* = 0.7039; (**c**) F (3,20) = 3.837, *p* = 0.0255; (**d**) F (3,20) = 5.692, *p* = 0.0055; (**e**) F (3,20) = 2.169, *p* = 0.1234; (**f**) F (3,20) = 4.596, *p* = 0.0133; (**g**) F (3,20) = 0.9823, *p* = 0.4209; (**h**) F (3,20) = 6.380, *p* = 0.0033.

Regarding the prefrontal cortex (Figure 8), MIA mice show a neuroinflammatory condition characterized by high levels of IL-1β and TNFα mRNA (panels d and f; *p* < 0.05) and of the microglia marker Iba1 (panel h; *p* < 0.01). Both PC1 and diclofenac treatment similarly counteract neuroinflammation (panels d, f, and h; *p* < 0.05). In this area, no alterations of PK2, PKRs, IL-6, and GFAP expression are evident (panels a–c, e, and g, respectively).

**Figure 9:**
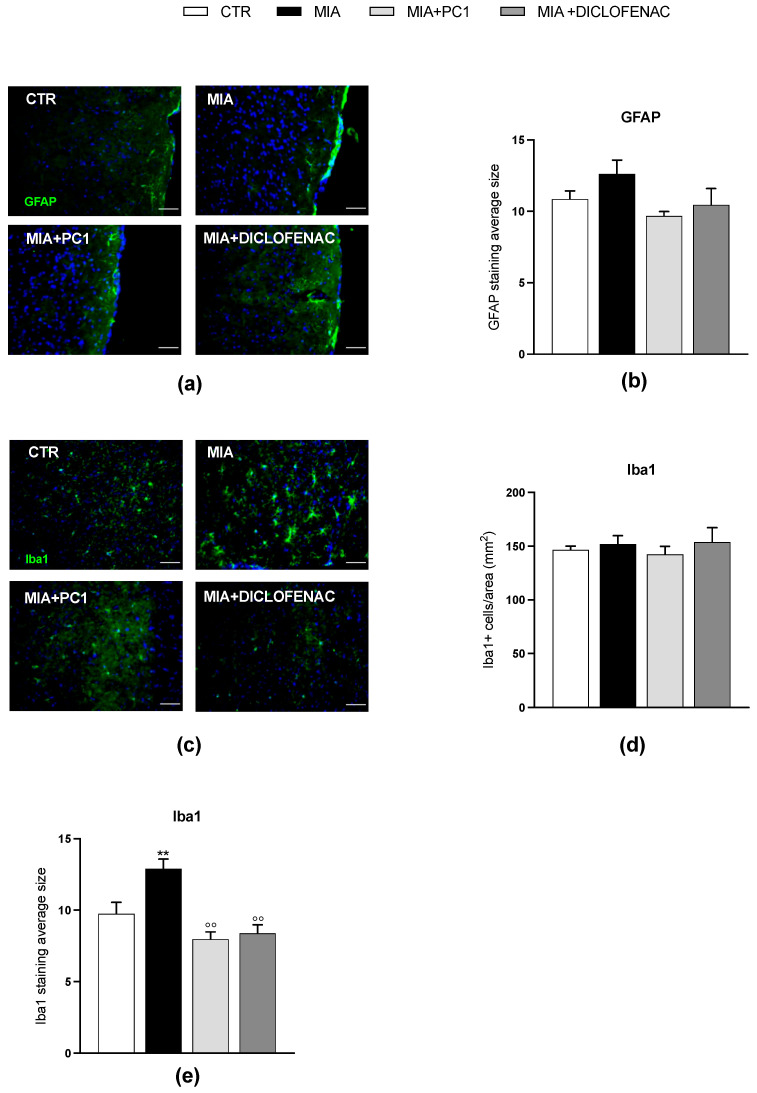
Effect of PC1 and diclofenac on microgliosis in the prefrontal cortex of OA mice: immunohistochemical analysis. Images of prefrontal cortex sections (day 28 post-OA) after IHC staining for GFAP (green; **a**) and Iba1 (**c**) have been converted to black and white and the “particle analysis” Fiji/ImageJ tool was then utilized to measure the average size of GFAP- and Iba1-positive particles (**b** and **e**, respectively). Representative images of prefrontal cortex sections after IHC staining for Iba1 (green)(**c**). The number of Iba1-positive cells/area has also been evaluated (**d**). Nuclei were labeled with the Hoechst33258 dye (blue). Scale bars: 50 µm. Data are the mean ± SEM of 3 animals/group. Statistical analyses were performed by means of One-way ANOVA followed by Tukey’s post hoc test. ** *p* < 0.01 vs. CTR; °° *p* < 0.01 vs. MIA. Treatments: (**b**) F (3,8) = 1.397, *p* = 0.3126; (**d**) F (3,8) = 0.3479, *p* = 0.7919; (**e**) F (3,8) = 11.69, *p* = 0.0027.

As shown in Figure 9, in line with qPCR results, no reactive astrogliosis is detected in the prefrontal cortex of MIA-exposed animals (panels a and b) since the immunostaining for GFAP displays no difference among the four experimental groups. Additionally, immunofluorescence analysis of Iba1-positive cell morphology shows hypertrophic and intensely stained cells (panel c) with a significantly increased average size in MIA animals (panel e, *p* < 0.01) with respect to the CTR group, thus confirming the development of microgliosis even in the absence of differences in the number of Iba1-positive cells (panel d). Both PC1 and diclofenac proved able to counteract MIA-induced microgliosis, as shown by the significant decrease in the average size of Iba1-positive cells (panel e; *p* < 0.01). Supplementary Figure S1, panel b, shows immunostaining negative control.

#### 3.5.3. TNFα Protein Levels in the Hippocampus and Prefrontal Cortex

As shown in Supplementary Figure S2, we also evaluated TNFα protein levels by ELISA assay in the hippocampus (panel a) and prefrontal cortex (panel b), fully confirming mRNA expression. Indeed, MIA mice are characterized by a significant increase in TNFα protein content in the hippocampus and prefrontal cortex (*p* < 0.001), and both PC1 and diclofenac counteracted MIA-induced effect (*p* < 0.001).

## 4. Discussion

Osteoarthritis, OA, is the most prevalent joint disease frequently characterized by the development of chronic pain and disability. OA pain is often related to the presence of mood alterations, such as anxiety and depression [10,11,12], which can aggravate pain perception, contributing to the development of chronic pain. Current analgesic strategies for the treatment of OA pain have modest effects and are often associated with severe side effects. Thus, OA pain treatment is still an unmet clinical need, which requires a better comprehension of the mechanisms underlying chronic pain. In this context, treatments that simultaneously control the nociceptive, affective, and cognitive manifestations could represent an efficient therapeutic strategy for chronic OA pain.

Our study suggests for the first time a role for the prokineticin (PK) system, a family of chemokine deeply involved in inflammation and pain [29,31,52], in local inflammatory and central sensitization processes as well as in mood alterations associated with pain in the monosodium iodoacetate mouse model of osteoarthritis.

Prokineticins (PK1 and PK2) and their receptors, PKRs, are implicated in the regulation of several biological functions [30]. Moreover, we and others deeply characterized the role of the PK system in inflammation as well as in the neuroinflammatory and sensitization processes that contribute to chronic pain establishment [37,43,45,53,54,55,56].

In this work, we used the MIA-induced OA mouse model, one of the most validated and used rodent models, to assess pain and its treatments [38,39]. MIA injection into the intraarticular space results in functional impairment that resembles human OA. MIA animals, indeed, develop pain-related behaviors, such as thermal hyperalgesia and mechanical allodynia [41,57,58]. In accordance with the literature, our results show that MIA mice develop mechanical allodynia in the ipsilateral paw; this symptom was already evident 7 days after MIA injection and maintained up to day 28. OA-induced allodynia is accompanied by motor deficits and fatigue-like behavior, a typical condition of OA patients. In addition, 28 days after MIA administration, in the presence of a fully developed and long-lasting pain symptomatology, OA mice also display anxiety- and depression-like behaviors. Our results are in line with recent preclinical studies in which MIA-treated animals exhibit psychiatric-like features in parallel with OA [15,16,17,18,19]. Our data clearly show that the chronic therapeutic treatment started 14 days after MIA injection, with both the PK system antagonist PC1 and the NSAID diclofenac significantly and similarly counteract pain hypersensitivity and improve motor deficits. Considering pain-like behavior, our results suggest that the antiallodynic effect of diclofenac is greater and faster than that of PC1. This difference might be due to the ability of diclofenac to block prostaglandin (PG) synthesis, thus preventing their direct sensitizing effect on nociceptors.

Previous studies in MIA-induced OA models suggested that the analgesic efficacy of NSAIDs varies over time. Specifically, these reports suggested that the inflammation contributing to pain in the early phase of OA (i.e., up to approximately 2 weeks after MIA injection) is sensitive to NSAIDs [59,60,61] while a non-inflammatory late phase is resistant to NSAIDs [62,63,64]. We started our treatments 14 days after the MIA injection, likely in a phase in between the switch from inflammatory to neuropathic pain in which the PG pathway still plays an important role in OA pain [44].

Although a direct action of PK system antagonists on COX enzymes has never been described, an interaction between PK2 and the eicosanoid system was suggested [65]. The heat nociceptive response to Bv8/PK2 was evaluated in COX1- and COX2-KO mice, and the authors demonstrated that COX1-KO mice were 20 times less sensitive to Bv8/PK2 than WT mice, suggesting the importance of COX-1 for nociceptor activation by PK2 [65]. The same authors also suggested the existence of a peptidergic (CGRP+) nociceptor population expressing both COX-1 and PKRs where PG may contribute to the excitatory effect of Bv8/PK2 [65].

The biochemical evaluations performed in the articular joint indicate the presence of an ongoing inflammatory/degenerative process. Indeed, 28 days after OA induction, we detected a marked upregulation of both PK2 and PKR1. These changes were also related to increased levels of pro-inflammatory cytokines and metalloproteinase (MMP) 13. MMP-13 is a primary catabolic enzyme involved in cartilage degradation through its specific ability to cleave type II collagen. The breakdown products of cartilage stimulate type A synoviocytes to release inflammatory cytokines such as TNFα, IL-1β, IL-6, and MMPs, which, in turn, enhance the catabolic effect on chondrocyte metabolism, thus accelerating the progression of OA [66,67]. Additionally, synoviocytes, together with infiltrating inflammatory cells, chondrocytes, as well as infrapatellar fat, may also express PKRs and produce PK2 [32,33], which can sustain an inflammatory loop in turn promoting the recruitment of inflammatory cells, the release of pro-inflammatory/pro-algogenic mediators and a direct sensitization of nociceptors [29,31]. Similarly to diclofenac, the treatment with the PKR antagonist PC1 can counteract the degenerative and inflammatory process in the knee joint by reducing both PK2 and PKRs as well as pro-inflammatory cytokines and MMP-13 overexpression. In accordance with our data, PK2 expression levels were significantly elevated in the joint of CIA (collagen-induced arthritis) mice, and this overexpression was correlated to arthritis severity. The administration of PKRA7, a PKR antagonist, suppressed the severity of arthritis and reduced IL-1β and lL-6 expression in the joint [32]. Similarly, in the same CIA model, a recent paper [34] demonstrated that the treatment with PC1 improved paw edema, pain, and locomotor activity. PC1 was also able to reduce plasma malondialdehyde (MDA) levels, thus reducing oxidative damage and decreasing the expression of PK2, PKRs, TNFα, IL-1β, CD4, CD8, and NF-kB in the joint [34]. In addition, both PK2 and PKRs were overexpressed in the synovial tissue of rheumatoid arthritis (RA) and OA patients [33], and we recently described high levels of PK2 in the synovial fluid of patients with both traumatic meniscal injuries and knee osteoarthritis [35]. In this scenario, sensitized neurons expand their receptive fields, spreading hypersensitivity from the knee to adjacent areas and reducing the mechanical threshold around the joint, a phenomenon observed in OA patients [2,7]. In this regard, MIA mice display allodynia in the ipsilateral paw and are characterized by a neuroinflammatory condition in the sciatic nerve and spinal cord. As we already observed in the MIA OA model at earlier time points, i.e., 14 [41] and 21 [49] days after MIA injection, we found a neuroinflammatory condition affecting the sciatic nerve and the spinal cord 28 days after OA induction as well. In both tissues, but particularly in the sciatic nerve, we observed a marked upregulation of PK2 and PKR1, accompanied by high levels of IL-1β and IL-6. At the nerve level, considering the upregulation of GFAP and CD11b, the neuroinflammatory condition could be due to the presence of activated Schwann cells or infiltrating macrophages as a consequence of the ongoing demyelination process [68], which could also explain the observed increase in ATF3. In the spinal cord, 28 days after MIA injection, the pro-inflammatory cytokine and PK system upregulation might be due to astrocytes. Indeed, at this time point, we did not register any alteration in microglia markers. Considering our previous papers, in which at both 14 and 21 days after OA induction, we found high levels of several microglia markers [41,49], we might speculate that 28 days after MIA, microglia has switched back to a homeostatic state and is no longer in a reactive one. As already observed in other models of chronic pain, the PK system is upregulated in both PNS and CNS, contributing to the onset and maintenance of chronic pain [37,43,45,53,54,55,56]. PK2 upregulation is induced through STAT3 activation that binds the enhancer site of PK2 promoter [69,70,71]. STAT3 activation by IL-6 and IL-1 signaling was recently demonstrated in spinal cord neurons and astrocytes [71,72]. PK2 may contribute to astrogliosis and to the production of pro-inflammatory cytokines, such as IL-1β and IL-6, which in turn stimulate astrocytes and neurons to induce further PK2 expression, thus suggesting the presence of a feed-forward loop. It was also suggested that the overexpression of PK2 and PKR2 on activated astrocytes can act as an astrocytic-autocrine-growth factor [30].

Our results show that PC1 treatment, similar to diclofenac, can counteract the neuroinflammatory condition in the sciatic nerve and spinal cord. Considering the effect of the drugs on pain, we might speculate that the effect could be in part due to their ability to counteract synovial inflammation and degenerative processes in the joint. The reduction in the direct sensitization effect of cytokines and PK2 on nociceptors might interrupt the central sensitization process, which is important in pain chronicization [6,57,73].

As already mentioned before, our results indicate that OA mice characterized by hypersensitivity and by the presence of a marked neuroinflammatory condition in PNS and CNS are also distinguished by the presence of anxiety- and depression-like behaviors. Previous studies suggested that persistent pain may trigger alterations in brain areas involved in affective responses, which over time may lead to emotional comorbidities, including anxiety- and depression-like behavior [15,16,17,18,19,74,75]. The presence of such behavioral alterations is related to a clear neuroinflammatory condition in brain areas, such as the hippocampus and prefrontal cortex, that are crucial for mood control [76,77] and are important regulators of the affective and emotional component of pain. Indeed, in the prefrontal cortex, we observed an increase in the mRNA levels of the pro-inflammatory cytokines IL-1β and TNFα, along with the upregulation of the microglia marker Iba1, without changes in GFAP expression. These alterations are also supported by specular changes in the protein content evaluated both by ELISA and immunofluorescence analysis, suggesting the presence of an ongoing microglia response in this brain area. In fact, Iba1-positive cells show an intense staining and a hypertrophic morphology with a significantly increased average size in MIA animals. These data confirm the presence of microgliosis even in the absence of differences in the number of Iba1-positive cells. As mentioned above, microglia usually precociously react to damage/stressful conditions, producing pro-inflammatory mediators such as cytokines, and its activation is usually followed by a secondary astrocyte response. In accordance with our data, alterations in microglial activity in the medial prefrontal cortex were associated with the presence of emotional alterations such as anxiety and depression in MIA OA mice [16,17]. Our results in the hippocampus suggest the presence of a more marked neuroinflammatory condition. Indeed, the increase in pro-inflammatory cytokines is also accompanied by increased levels of PK2 and PKR2, the receptor subtype mainly expressed in the brain [30]. Differently from the prefrontal cortex, in the hippocampus of MIA OA mice, we found clear astrogliosis, as demonstrated by the increase in the average size of GFAP-positive cells and by higher GFAP mRNA levels, whereas no signs of microgliosis were detected.

The results obtained in the two brain areas let us hypothesize the presence of a time lag in the development of neuroinflammation between the hippocampus and the prefrontal cortex. In the prefrontal cortex of OA mice characterized by the development of anxiety- and depression-like behavior, the neuroinflammatory condition is probably at early stages and mainly due to reactive microglia, while in the hippocampus, a more advanced and marked neuroinflammation is observed, mainly due to reactive astrogliosis which may be responsible for PK system alterations. In the hippocampus, at this time point, microglia is probably back to a homeostatic state. However, the expression of PKRs in this cell population allows them to react to PK2 paracrine action, thus sustaining a neuroinflammatory condition.

In a mouse model of bortezomib-induced neuropathic pain, we recently demonstrated that long-lasting pain was correlated to mood alterations and accompanied by increased levels of PK2 and its PKR2 in the hippocampus, together with a general neuroinflammatory condition in the hippocampus, prefrontal cortex, and hypothalamus [37]. In addition, it is widely demonstrated in the literature that following different brain insults (e.g., hypoxia, ischemic damage, neurodegenerative diseases, toxicity), PK2 and PKR2 are usually overexpressed by both neurons and astrocytes in specific hippocampal areas and in cortical neuron cultures [78,79,80,81]. It was also suggested that the overexpression of PK2 and PKR2 on astrocytes can represent an astrocytic-autocrine-growth factor [30]. Therefore, we might speculate that, in this area, astrocytes could be responsible for PK system upregulation in the OA model. However, we are aware that colocalization immunofluorescence experiments could have clarified this aspect, and we are therefore conscious of this limitation of the work.

Our results clearly show that the treatment with PC1 efficaciously counteracts the presence of anxiety- and depression-like behaviors in OA mice, while diclofenac seems to be mainly effective on anxiety. The effect of the two drugs on neuroinflammation seems similar, even if only PC1 can effectively decrease the overexpression of PKR2 and PK2 in the hippocampus. These results might probably explain the lower effect of diclofenac on depression-like behavior. It was indeed demonstrated a direct role of PK2 in mood regulation [36,37]. Intracerebroventricular injection of PK2 into mice leads to an increase in anxiety- and depression-like behavior, while PK2-KO mice display reduced anxiety and depression [36]. Our immunofluorescence results also underline that both drugs were only partially able to counteract reactive astrogliosis. Considering that both drugs were able to significantly reduce GFAP mRNA, we could speculate that the effect of the drugs on protein could be observed at later time points.

In conclusion, our data demonstrate the role of the PK system in the inflammatory and neuroinflammatory pathways involved in OA pathophysiology and pain and in its comorbid affective manifestations. We can suggest that the effect of PC1 in supraspinal areas and on behavior can be firstly due to its ability to counteract peripheral inflammation and degenerative processes in the joint. The reduction in the sensitization effect of cytokines and PK2 on nociceptors might interrupt the central sensitization process, which is important in pain chronicization and supraspinal activation. The reduction in the inflammatory flow from PNS and the spinal cord could, in part, explain the alteration in cytokines and microglia markers observed in the prefrontal cortex without changes in the PK system. On the other hand, considering that PC1 can pass BBB, we can not exclude a direct effect of PC1 on PKR expressed by glial cells in supraspinal areas. Our results suggest, however, that both PKR antagonist PC1 and diclofenac counteract peripheral and central PK system activation, reduce pain and neuroinflammation, and contrast the development of mood alterations. The ability of PC1 to reduce the PK system in the hippocampus makes it more effective in depression-like behavior, suggesting the importance of the PK system in the development of this condition. NSAIDs, such as diclofenac, represent the first-line treatment against OA; however, their well-known gastrointestinal and the more recently recognized cardiovascular side effects are a cause of concern in many patients [82]. On the other hand, although the antagonism of the PK system has never been tested in clinical trials, the many preclinical studies with PK system non-peptidic antagonists demonstrated their safety and the absence of toxicity at anti-inflammatory and analgesic dosage [34,37,45,56,83]. These findings identify the PK system as a promising target to control OA pain and its complications.

## Data Availability

The datasets used and/or analyzed during the current study are available from the corresponding author on reasonable request.

## Supplementary Materials

The following supporting information can be downloaded at: https://www.mdpi.com/article/10.3390/cells12182255/s1, Figure S1: Specificity of Iba1 and GFAP immunostaining; Figure S2: Effect of PC1 and diclofenac on TNF alpha protein content in the hippocampus and prefrontal cortex of OA mice.
