# Supplementary material for: Prokineticin System Is a Pharmacological Target to Counteract Pain and Its Comorbid Mood Alterations in an Osteoarthritis Murine Model
Source: Cells. 2023 Sep 12;12(18):2255. doi: 10.3390/cells12182255 (PMC10526764; doi:10.3390/cells12182255)
Supplement: Supplementary file 1 [file cells-12-02255-s001.zip › cells-2528931-supplementary.pdf]

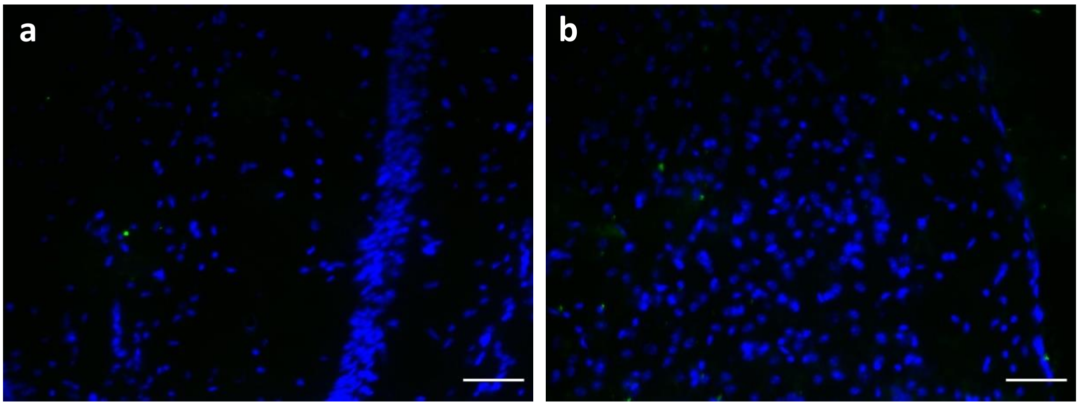

Supplementary Figure S1 | Immunostaining negative control. Pictures show immunostaining negative control in the hippocampus (a) and prefrontal cortex (b), where primary antibodies (anti-Iba1 and anti-GFAP) were omitted and sections were incubated with secondary antibody (AlexaFluor® 488-conjugated goat anti-rabbit) only. Nuclei were labeled with the Hoechst33258 dye (blue). Scale bars: 20 mM.

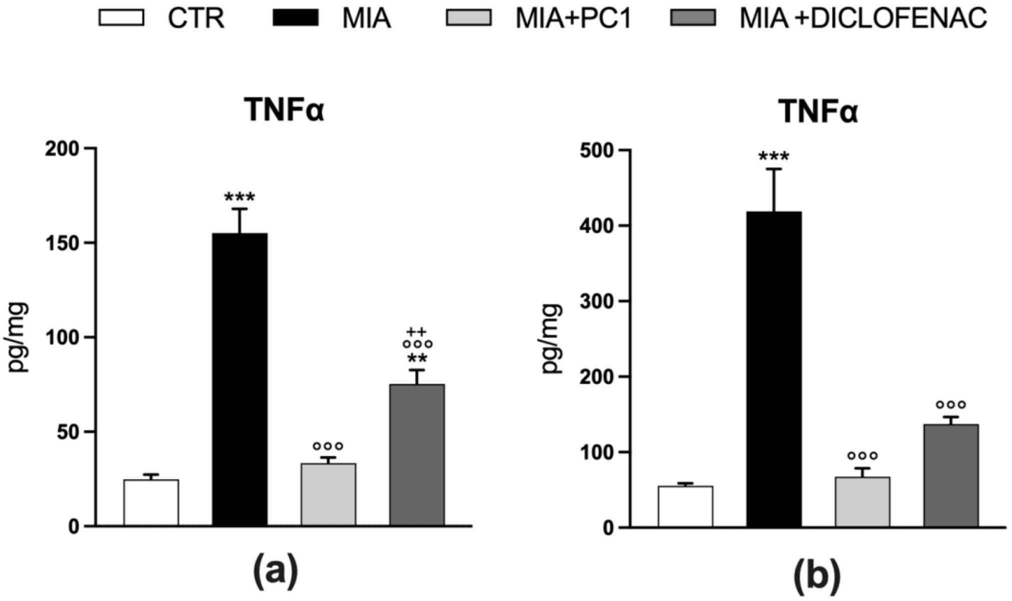

Supplementary Figure S2| Effect of PC1 and diclofenac on TNF alpha protein content in the hippocampus and prefrontal cortex of OA mice. Protein expression levels of TNF*α* were evaluated (ELISA method) in the hippocampus (a) and prefrontal cortex (b) (day 28 post OA). Results are expressed as mean ± SEM of 5 animals/group. Statistical analyses were performed by One-way ANOVA followed by Tukey's post-hoc test. $^{**}p < 0.01$, $^{***}p < 0.001$ vs CTR; $^{°°°}p < 0.001$ vs MIA; ++ $p < 0.01$ vs MIA+PC1. Treatments: (a) $F(3, 16) = 60.70$, $p<0.0001$; (b) $F(3,16) = 34.01$, $p<0.0001$.
